# A pharmacometrician’s role in enhancing medication use in pregnancy and lactation

**DOI:** 10.1007/s10928-020-09707-y

**Published:** 2020-08-16

**Authors:** Sara K. Quinney, Peter L. Bonate

**Affiliations:** 1 Department of Obstetrics and Gynecology, Division of Clinical Pharmacology, Department of Medicine, Center for Computation Biology and Bioinformatics, Indiana University School of Medicine, Indianapolis, IN USA; 2 Astellas, 1 Astellas Way, Northbrook, IL 60062 USA

Over 200 million pregnancies occur worldwide annually [1], yet pregnant women have historically been excluded from clinical studies. However, women who are pregnant are not immune to chronic disease (e.g. asthma, seizure disorders) or acute illnesses (e.g. viral infections or injuries). Additionally, pregnant women suffer from conditions associated with the pregnancy such as gestational diabetes or hypertension. Many women therefore need medication treatment during their pregnancy. Over 68% of women report taking at least one prescription drug, excluding vitamins, during their pregnancy, with a large proportion of women taking for or more drugs [2].

Prior to the early 1990s, women were largely underrepresented in clinical research, and pregnant women were summarily excluded from participation in clinical trials [3]. In fact, if a woman enrolled in a drug trial became pregnant during the course of the study, she was often removed from the study drug immediately. However, physiology differs between women and men, and between nonpregnant and pregnant women, leading to differences in drug distribution and response. It is difficult to extrapolate the effects of a drug that are observed in men to pregnant women. The National Institutes of Health Revitalization Act of 1993 required that women be included in NIH-funded studies unless it is inappropriate to the health of the subject or purpose of the research [4]. The Institute of Medicine’s committee on Women and Health Research unanimously asserted that “pregnant women be presumed to be eligible for participation in clinical studies.” [3] The committee also debated whether pregnant women should only be excluded from participation in research when there is no likelihood of medical benefit in pregnant women or when risk of harm to the fetus is known or can be inferred from preclinical studies. In spite of this, many Institutional Review Boards (IRB’s) continue to view pregnancy as an automatic reason to exclude women from clinical trials. This practice has left pregnant women as clinical orphans.

Pharmacometrics, including pharmacokinetic/pharmacodynamic modeling, systems pharmacology, and machine learning approaches, provides a valuable tool set for optimizing drug therapy in pregnant women. This themed issue of *The Journal of Pharmacokinetics and Pharmacodynamics* focuses on the challenges of conducting drug studies in pregnant and lactating women and the opportunities for pharmacometricians and data scientists to enhance research in this area. McKiever et al. provide a historical background of inclusion of pregnant women in clinical studies from initial experiences with diethylstilbestrol (DES) and thalidomide, which led to legislative changes excluding women of reproductive age from early phase clinical trials, to current philosophy recognizing the importance of well-conducted clinical studies in this population [5]. They also provide an example of repurposing pravastatin, a previously Category X medication, for prevention of preeclampsia. This case also demonstrates the significance of the FDA’s pregnancy labeling change which replaced the categorical labeling system with more detailed information on studies in pregnancy.

Physiologically based pharmacokinetic (PBPK) modeling is a valuable tool for assessing changes in pharmacokinetics during pregnancy. PBPK models provide a means of incorporating physiological changes across pregnancy with knowledge of a drug’s ADMET characteristics to predict changes in PK across gestation. In this issue, Kazma et al. review anatomical and physiological changes in pregnant women and how they can be used to inform PBPK models [6]. Abduljalil et al. provide examples of how PBPK modeling can inform drug dosing in pregnancy and estimate fetal drug exposure [7]. They also provide examples of individualizing dosing based upon additional covariates, such as pharmacogenomics or drug-drug interactions.

The maternal-infant connection does not end at delivery. 95% of women worldwide breastfeed their infants at least once, and 40–60% of infants are breastfed for the first 2 years of life [8]. The American Academy of Pediatrics recommends exclusive breastfeeding for the first 6 months of life [9]. With no regulatory requirement to conduct drug studies in lactating women, there is a paucity of data on pharmacokinetics and safety of drugs in this population. The complexity of maternal and infant physiological changes postpartum, including variability in milk production and infant feeding patterns complicate pharmacokinetic studies in breastfeeding. In this issue, Anderson and Momper describe current best practices in pharmacokinetic studies in lactating women and estimation of infant dose [10]. They also describe challenges in conducting clinical lactation studies and limitations to the use of pharmacokinetic modeling approaches, including integration with in utero exposure and limited knowledge of postpartum physiological changes.

The burgeoning field of artificial intelligence (AI), including machine learning, has shown promise in optimizing drug use in a number of areas including oncology, diabetes and anesthesia [11–13]. Yet, few studies have applied AI to pharmacological studies in pregnancy and lactation. Davidson and Boland conducted a systematic review of literature relating to AI in pregnancy [14]. They identified studies relating to obtaining data from clinical records, clinical decision support focusing on diagnosis and disease management, and prediction of birth outcomes were identified. However, relatively few papers incorporated pharmacological data. Harnessing the potential of AI to enhance pharmacological therapy in pregnancy and lactation has great promise to inform medication safety and efficacy for both mother and child.

Great progress has been made in the field of obstetric clinical pharmacology over the past decade, thanks in part to new FDA Guidances [15, 16] and NIH funding initiatives, including the Obstetric Pharmacology Research Centers [17]. While there has been some growth in obstetric pharmacology over the past decade, the number of studies published regarding pharmacokinetics in pregnancy and lactation remains abysmal. A search of Pubmed for “pharmacokinetics” and “pregnancy” clinical trials returns less than 40 publications a year; the number of lactation studies is in the single digits. Clinicaltrials.gov lists only 77 active or planned studies including the terms “pregnancy” and “pharmacokinetics”, with less than half of those receiving support from pharmaceutical industry. Yet, every individual alive today has been affected by pregnancy. With the large majority of pregnant women taking medication, it is critical that we understand how pregnancy alters the pharmacokinetics of drugs both for optimized treatment of maternal conditions and safety of the developing fetus. The advantages of breastfeeding are numerous [9], yet women will often discontinue breastfeeding due to fear of how a drug may affect their child [18]. Often these concerns are unfounded. However, with the paucity of studies providing information on drug exposure to breastfed infants women err on the side of “safety”, while inadvertently withholding a valuable resource from their infant.

As discussed in a recent New England Journal of Medicine perspective, more work is needed to “get it right” [19]. What is missing from the New England Journal perspective is the role of quantitative pharmacology approaches to augment study design and analysis of the often sparse data available from clinical obstetric studies. Pharmacometrics, systems pharmacology, artificial intelligence, and other novel quantitative approaches provide important tools to explore the pharmacokinetics and pharmacodynamics of drugs in pregnancy and lactation while minimizing risk to mother and baby.

Including pharmacokinetic sampling in studies of pregnant and lactating women should become the rule, not the exception. It is critical for clinical pharmacologists and pharmacometricians to be consulted early in protocol development. Within the department of one of this editorial’s authors (Quinney), she has been asked to evaluate drug concentrations in samples that have been previously collected. Often, she finds that the study design is inadequate or that the addition of one or two well-timed sample collections would have greatly magnified the knowledge gained. Linking risk to medication use during pregnancy also requires a more granular level of data than is typically collected in studies. Large prospective cohort studies of pregnant and lactating women should strive to collect well-characterized medication use data during pregnancy and lactation, including information on timing and dosing. Asking “what medications have you taken?” will not provide information on how much medication was taken and when. Investigators should leverage use of electronic health records, wearables, and other information resources through integrative pharmacometric and machine learning approaches to enhance understanding of pharmacotherapy in pregnancy and lactation. While there has been an upsurge in PBPK modeling in obstetrics, additional studies are required to validate the assumptions of these models and further understand sources of inter- and intra-individual variability.

Finally, in order to benefit patients, results of studies must be implemented into widespread clinical practice. A survey of American Academy of Allergy, Asthma & Immunology membership found that less than half of clinicians were not aware of the FDA’s pregnancy and lactation labeling changes and 95% continued to use the pregnancy letter categories [20]. This indicates the difficulty in disseminating new knowledge to stakeholders. Pharmacometricians should work in tandem with the informatics and medical communities to develop clinical decision support systems to move knowledge gained from obstetric and lactation pharmacology research to clinicians and patients.

Research in obstetric pharmacology has advanced greatly over the past decades. However, much work remains to be done. By exploiting tools commonly applied to other diseases, pharmacometricians can play a significant role in optimizing drug therapy in pregnant and lactating women. Not only is the health of women at stake, but also the health of future generations.
